# Surgical Technique for Stroke Prevention During Ventricular Assist Device Removal

**DOI:** 10.1016/j.jaccas.2025.105725

**Published:** 2025-11-19

**Authors:** Hannah Copeland, Tanisha Rajah, David Blitzer, John Morton

**Affiliations:** a Division of Cardiothoracic Surgery, Community Heart and Vascular Hospital, Indianapolis, Indiana, USA; b Birmingham Medical School, University of Birmingham, Birmingham, United Kingdom; c Division of Cardiac, Thoracic, and Vascular Surgery, Department of Surgery, New York Presbyterian-Columbia University Irving Medical Center, New York, New York, USA; d Division of Cardiothoracic and Vascular Surgery, Lutheran Hospital, Indiana University School of Medicine - Fort Wayne, Fort Wayne, Indiana, USA

**Keywords:** cerebroprotection, heart failure, Impella, left ventricular assist device

## Abstract

Mechanical circulatory support with the Impella 5.5 is increasingly used as a bridge to heart transplantation or durable left ventricular assist device in patients with chronic heart failure. However, thrombosis and stroke remain significant concerns upon Impella 5.5 device removal. To mitigate this risk, one technique involves placing the patient in the Trendelenburg position, temporary carotid occlusion, and Fogarty thrombectomy during device explantation. We retrospectively report outcomes from 3 patients (mean age 45; 2 male) with chronic end-stage heart failure who underwent this technique between September 2022 and June 2023. Impella implantation was via axillary access as a bridge to heart transplant (n = 2) or durable left ventricular assist device (n = 1), with device removal 13 to 43 days later. All patients remained neurologically intact postoperatively, with no strokes or deficits. This case series supports the feasibility and safety of this technique in mitigating stroke risk during Impella 5.5 removal in high-risk surgical candidates.

## Graphical Abstract

**Figure:**
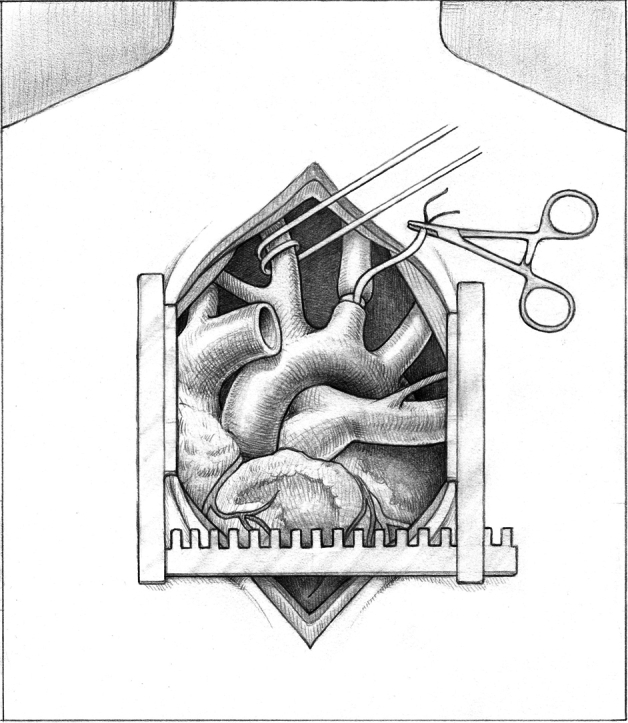

Mechanical circulatory support has been increasingly used as a bridge to orthotopic heart transplant.^1^ Embolization and stroke are well-documented complications of left ventricular assist device (LVAD) removal,^2^ with the incidence of cerebrovascular accidents rates reaching 7.4% in specific patient populations.^3^ We describe a technique for preventing embolic stroke during Impella 5.5 (Abiomed) removal in heart transplant or durable LVAD implantation.

**Take-Home Messages**

• Bilateral carotid occlusion, followed by Fogarty thrombectomy is a simple, effective method to prevent stroke during Impella 5.5 removal.
• No strokes occurred in all 3 patients using this technique.

## Technique

**Figure 1:**
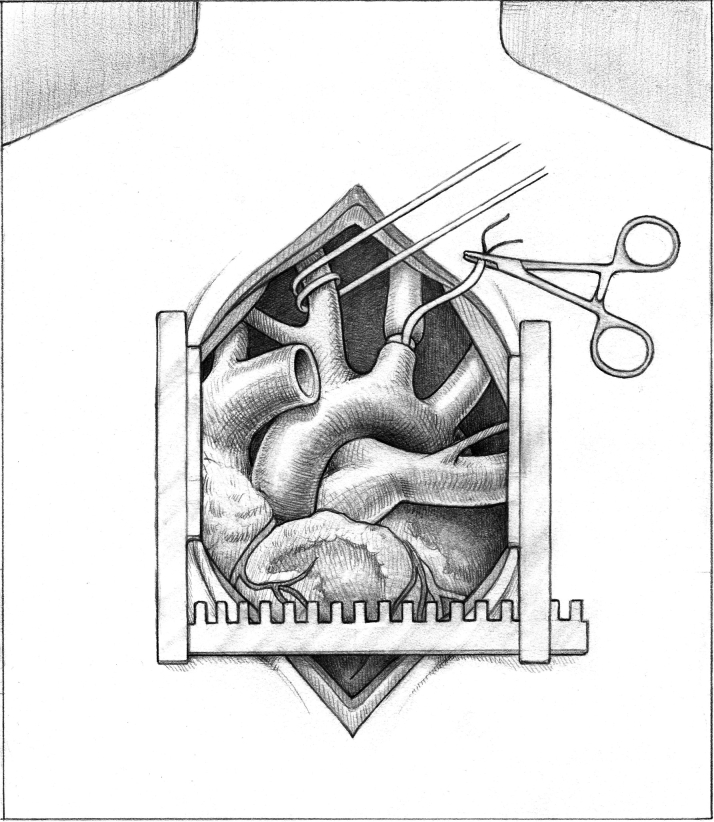
Umbilical Tapes Placed Around the Bilateral Carotid Arteries

The 8-mm graft for the axillary artery anastomosis is identified and prepared. A median sternotomy is performed, and hemostasis is achieved. The pericardium is opened and retracted. First the head vessels are palpated to ensure there are no palpable plaques. The right carotid artery past the innominate artery bifurcation and the left carotid artery are identified and encircled with umbilical tapes or vessels using a right angle (Figure 1). This is followed by intravenous heparin administration. Cannulation sutures are then placed in the ascending aorta and either the right atrium (for durable LVAD) or bicaval for heart transplantation. Once an appropriate activated clotting time is confirmed, the ascending aortic and the venous cannulae are placed, secured, and de-aired. The Impella 5.5 is gradually weaned and turned off, and cardiopulmonary bypass is initiated.

**Figure 2:**
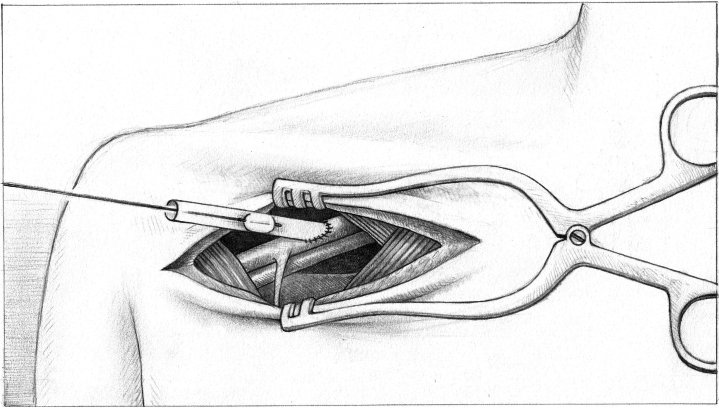
Demonstration of the Balloon Pull During Thrombectomy

The patient is then placed in the Trendelenburg position. The right and left carotid arteries are snared, and either: 1) the Impella 5.5 is removed completely in the setting of durable LVAD implantation; or 2) the aortic cross-clamp is applied in the setting of heart transplant. The Impella is cut at the level of the aortic cross clamp, followed by the removal of the remaining Impella 5.5 in heart transplantation. The axillary graft is clamped after the removal of the Impella; the clamp is then removed off the axillary graft, and a Fogarty thrombectomy of the graft and axillary artery are performed with removal of clot and debris (Figure 2). To perform the thrombectomy, a Fogarty catheter is passed through the graft, the balloon is inflated and pulled back, and the clot is removed. The Fogarty catheter is passed into the axillary artery. Immediately afterwards, the snares are released and removed, leaving the right and left carotid arteries open. The axillary graft is cut and oversewn with a 4-0 prolene running suture, and the wound is then packed and closed at the end of the procedure.

## Results

In this case series, we reviewed 3 patients who had Impella 5.5 removal between September 2022 and June 2023 using this novel technique. All 3 patients had chronic end-stage heart failure due to varying etiologies. Implantation of the Impella 5.5 was indicated either as a bridge to durable LVAD (n = 1) or a bridge to heart transplantation (n = 2). Impella insertion was via axillary access, with removal 13 to 43 days later.

Two out of 3 patients were male, with the average age being 45 years. In all 3 cases, no strokes occurred, with all patients being alert and orientated postoperatively. A summary of the case series is presented in Table 1.

**Table 1:** Summary of Case Series Using Novel Technique for Stroke Prevention During Impella Removal

Case	Patient Demographics	Heart Failure Etiology	Indication for Impella	Duration of Impella Implantation	Thromboendarterectomy Required?	Postoperative Length of Stay	Postoperative Neurological Findings
1	Female Age 24 years	Kawasaki’s vasculitis, ischemic cardiomyopathy	Bridge to LVAD	13 days	No	12 days	No focal deficits
2	Male Age 46 years	Dilated cardiomyopathy	Bridge to OHT	27 days	Yes	19 days	No focal deficits
3	Male Age 66 years	Mixed (ischemic cardiomyopathy/non-ischemic cardiomyopathy)	Bridge to OHT	43 days	Yes	16 days	No focal deficits

LVAD = left ventricular assist device; OHT = orthotopic heart transplant.

## Discussion

Current strategies to prevent thromboembolic complications during Impella 5.5 removal vary across centers. While many experienced centers may adopt similar techniques, no standardized approach has been established, and the literature on this topic remains limited.

One approach is the use of embolic protection devices (EPDs); Ranganath et al^4^ reported successful use of these in a patient undergoing Impella removal without anticoagulation. However, some studies suggest that the use of EPDs has been associated with an increased risk of complications due to the required manipulation of the device within diseased aortic and carotid vessels.^5^ In addition, EPDs require radial or femoral artery access for their deployment, adding procedural complexity. Comparatively, our technique is a less-invasive form of embolic protection with minimal associated risks.

In contrast, our technique of temporary bilateral carotid artery occlusion achieves the same goal of embolic protection through a less-invasive approach. We use near-infrared spectroscopy monitoring throughout the snaring of the vessels, which takes typically about 1 to 2 minutes in total. This technique protects the brain and right arm vessels from debris that could migrate to these areas while the Impella is removed as it passes along the aortic arch. Thrombendarterectomy was performed on all patients when we bridged from Impella to durable LVAD and/or heart transplant.

Temporary bilateral carotid artery occlusion also avoids the need for additional arterial access and minimizes manipulation within the vasculature, thereby reducing the risk of access-related and embolic complications. While many experienced centers may already employ similar methods, we believe that formal description and standardization of this technique can support wider adoption and improve procedural safety.

### Data Availability Statement

Data available upon request.

## Funding Support and Author Disclosures

The authors have reported that they have no relationships relevant to the contents of this paper to disclose.
